# Prognostic Relevance of BRCA1 Expression in Survival of Patients With Cervical Cancer

**DOI:** 10.3389/fonc.2021.770103

**Published:** 2021-11-08

**Authors:** E Sun Paik, Chi-Son Chang, Ye Lin Chae, So Young Oh, Sun-Ju Byeon, Chul Jung Kim, Yoo-Young Lee, Tae-Joong Kim, Jeong-Won Lee, Byoung-Gie Kim, Chel Hun Choi

**Affiliations:** ^1^ Department of Obstetrics and Gynecology, Kangbuk Samsung Hospital, Sungkyunkwan University School of Medicine, Seoul, South Korea; ^2^ Department of Obstetrics and Gynecology, Samsung Medical Center, Sungkyunkwan University School of Medicine, Seoul, South Korea; ^3^ Department of Pathology and Translational Genomics, Samsung Medical Center, Sungkyunkwan University School of Medicine, Seoul, South Korea; ^4^ Department of Pathology, Hallym University Dongtan Sacred Heart Hospital, Hwaseong, South Korea; ^5^ Department of Obstetrics and Gynecology, College of Medicine, Konyang University, Daejeon, South Korea

**Keywords:** BRCA1, gene signature, cervical cancer, immunohistochemistry, prognosis

## Abstract

**Objective:**

BRCA1 expression can be lost by a variety of mechanisms including germline or somatic mutation and promotor hypermethylation. Given the potential importance of BRCA1 loss as a predictive and prognostic biomarker in several cancers, the objective of this study was to investigate BRCA1 expression using immunohistochemistry (IHC) in cervical cancer and its possible prognostic relevance.

**Methods:**

Seventy patients with cervical cancer were enrolled in this study. Samples from each tumor were stained for BRCA1 and reviewed independently by gynecologic pathologists blinded to the BRCA status. Kaplan–Meier methods were used to estimate overall survival according to BRCA1 expression. Differentially expressed genes (DEGs) by BRCA1 expression were selected using GSE44001 dataset, which included 300 samples treated with radical hysterectomy. In addition, cox regression analysis with backward elimination was performed to select independent prognostic markers. Gene set enrichment analysis (GSEA) was done using these DEGs.

**Results:**

BRCA1 IHC was positive in 62.9% (44/70) of cases. Patients with BRCA1 expression showed better overall survival (100% *vs*. 76.2%, HR 0.20, 95% CI 0.04 – 0.99, p = 0.028) than those without BRCA1 expression. Analysis of gene expression profiles according to *BRCA1* expression identified 321 differentially expressed mRNAs. Gene set enrichment analysis results showed two dysregulated pathways (VEGF_A_UP.V1_DN and E2F1_UP.V1_UP). Of these DEGs, alterations of 20 gene signatures were found to be independently associated with survival outcomes of patients.

**Conclusions:**

BRCA1 expression in cervical cancer tissue is associated with survival. In addition, the identification of specific gene alterations associated with BRCA1 expression could help to provide individualized prediction in these patients.

## Introduction

Cervical cancer is the fourth most frequent malignancy in women and the seventh most common cancer overall worldwide (1). In Korea, it is one of common gynecologic cancers, accounting for 9.8% of newly diagnosed malignancies in women (2). To treat invasive cervical cancer, surgical resection and/or radiation and cisplatin-based chemotherapy are commonly used. After recurrence, there are only a few feasible treatment options. The prognosis of patients with recurrence of cervical cancer is generally poor (3, 4). Most cervical cancer patients become resistant to treatment. The resistance is either intrinsic or acquired during treatment (5). Chemoradiotherapy may induce DNA double-strand break (DSB), which is considered a lethal form of DNA damage. DNA damage can induce a series of molecular responses responsible for the maintenance of genome integrity (6). Deficiencies in DSB response and repair could lead to intrinsic resistance which will affect the prognosis of patients.

Loss of BRCA1 function in cancer cells can lead to absence of intact homologous recombinant DNA repair, and result in cells being more sensitive to agents that cause DSBs. BRCA1 loss is known to be associated with the loss of heterozygosity (7) or promotor hypermethylation (8, 9). It has been shown that miRNA regulation of BRCA1 mRNA stability might contribute to BRCA1 silencing (10, 11). A method for identifying BRCA1 expression would be useful for prognostication of cervical cancer patients. However, studies on genomic profiles of cervical cancer patients regarding BRCA1 expression are insufficient.

Despite recent decrease in cost and improved efficiency of sequencing, genetic testing is still expensive and time-consuming. Another alternative for assessing genetic implications in cancer is by identifying pathological features based on protein expression levels using immunohistochemical (IHC) analysis (12, 13). In this study, we performed BRCA1 expression analysis using immunohistochemistry (IHC), an inexpensive and widely available technique, in cervical cancer patients for its prognostic significance, and determined the correlation between its mRNA and protein expression. In addition, we identified genes associated with BRCA1 expression in cervical cancer to determine its clinical significance.

## Materials And Methods

### Patients

We analyzed data of 70 cervical cancer patients treated at the Department of Gynecologic Oncology, Samsung Medical Center from 2002 to 2009. Patients with rare histology or an advanced stage treated primarily with radiotherapy or chemotherapy were excluded. The tissue specimens and medical records were collected after we obtained informed consent from included patients and approval from the institutional review boards (IRB) of Samsung Medical Center, Seoul, Republic of Korea (approval no. 2009-09-002-002 and 2015-07-122). All of included patients had undergone radical hysterectomy with or without pelvic/para-aortic lymph node dissection as primary treatment. According to pathologic reports, patients with risk factors such as positive resection margin, lymph node metastasis, parametrial involvement, and stromal invasion of more than half of the cervix received adjuvant radiation treatment with or without a concurrent chemotherapy. After primary and adjuvant treatment, patients were routinely followed up every 3 months for the first 2 years, every 6 months for the next 3 years, and every 12 months thereafter. Progression-free survival (PFS) was defined as the time from the initial surgical treatment to recurrence or last follow-up. Overall survival (OS) was defined as the time from the initial surgical treatment to death or last follow-up.

### Immunohistochemistry (IHC) and Quantitative Evaluation

IHC was performed for FFPE 4-μm thick tissue sections using a standard protocol. Tissue blocks were used for routine pathological evaluations. Original archived hematoxylin-eosin–stained slides were reviewed by a pathologist. Following rehydration and deparaffinization of tissue sections, antigen retrieval for 20 minutes using 0.01 M citrate buffer (pH 6.0) and a boiling process (pressure cooker) was performed. Endogenous peroxidase block was done with H2O2 (3%). Incubation of sections was done with primary antibodies at 20°C (room temperature) for 30 minutes in a humid chamber. IHC staining was done using a primary monoclonal antibody for BRCA1 (Bio-Vision Inc., OH, USA, clone#3364-100) at a dilution of 1:400. Sections were sensitized and then incubated with a secondary antibody. The peroxidase reaction development was done using diaminobenzidine tetrachloride (DAB) as a chromogen. Counterstain of sections was done with hematoxylin.

**Figure 1:**
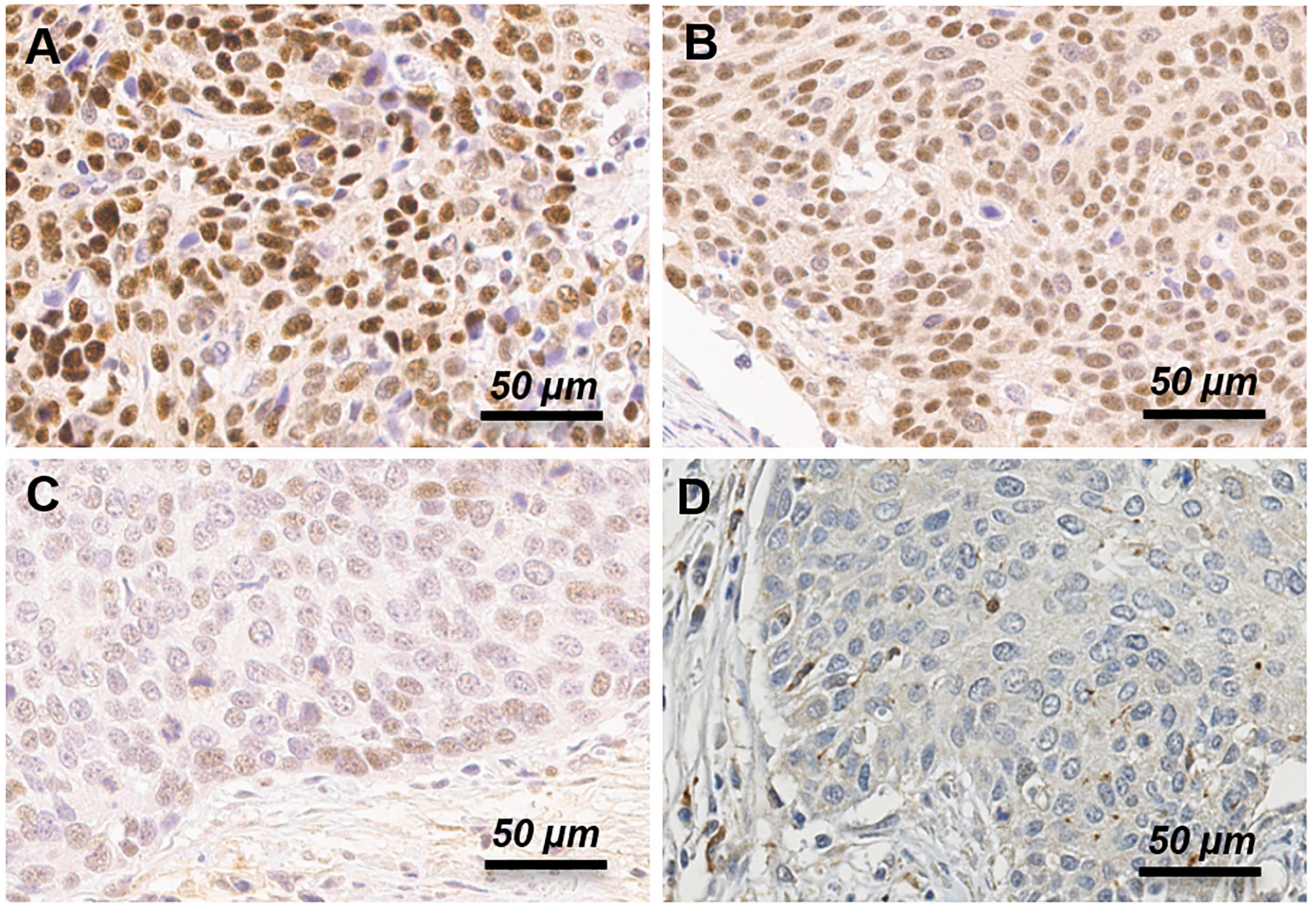
Immunohistochemistry expression of BRCA1 protein in uterine cervical cancer patients. Representative examples of strong **(A)**, moderate **(B)**, weak **(C)** and negative **(D)** expression of BRCA1 protein. Moderate and strong intensity grade was considered as positive, and weak and negative intensity grade was considered as negative for BRCA1 expression.

Staining results included the percentage of positive cells and intensities of stained cells in the nucleus and cytoplasm. IHC score was graded visually by pathologists. The staining intensity grade was divided to 4 groups (negative, weak, moderate, or strong). For avoidance of bias, the IHC score grading was performed independently by two pathologists, and quality-control was done by score comparison for equivalence (14). Moderate or strong intensity grade was considered as positive for BRCA1 expression (**Figure 1**). In case of discrepancy in judgement, conclusion was drawn by discussion using a multi-head microscope.

### *In Silico* Analysis for GSE44001 for the Selection of Differentially Expressed Genes According to BRCA1

To examine the prognostic significance of BRCA1 expression, the Gene Expression Omnibus (GEO) data were analyzed (15, 16). From a total of 300 available patient samples of GSE44001 (http://www.ncbi.nlm.nih.gov/geo/query/acc.cgi?acc=GSE44001), forty-nine samples were used in the IHC analysis of current study. For mRNA analysis, cDNA mediated annealing, selection, extension, and ligation (DASL) assay data were used (15). Obtained data were dichotomized by the distribution of expression of genes.

To identify differentially expressed genes according to BRCA1 expression, normalized expression levels were used. Differences in expression were featured by correlation coefficient (> 0.5 or < -0.5) and associated p*-*value (FDR-adjusted p < 0.05) in Pearson correlation analysis.

### Gene Set Enrichment Analysis (GSEA)

To determine pathways associated with BRCA1 expression, gene set enrichment analysis (GSEA) was performed using R package (GSEA.1.0.R) (17, 18). Curated oncogenic signatures v5.2 (including 189 gene sets) (https://www.gsea-msigdb.org/gsea/msigdb/genesets.jsp?collection=C6) were chosen to perform enrichment analysis among the two groups. Gene sets were first preprocessed to exclude those with < 10 or > 500 genes. The phenotype label was set as BRCA-high *vs*. BRCA-low. The t-statistic mean of genes was computed for each pathway using a permutation test with 1000 replications. For discovery, gene sets with a normalized p-value < 0.05 were chosen as significantly enriched.

### Statistical Analysis

For all statistical analysis, R software, version 3.1.3 (R Foundation, Vienna, Austria; http://www.R-project.org) was used. Student’s t-test or Mann–Whitney U-test was used for analysis of expression levels of proteins according to clinicopathological characteristics. Analysis using Spearman’s rho coefficient was performed for assessing correlations between proteins and mRNA expression levels. Survival distributions were estimated using the Kaplan–Meier method. The log-rank test was used for analyzing the relation of survival and each parameter. A Cox proportional hazards model was used for identification of independent predictors of survival. A p-value of < 0.05 was considered as significant.

## Results

### Clinicopathological Characteristics of Patients (Patient and Tumor Characteristics)

We analyzed 70 patients with cervical cancer. In this patient group, thirteen cases developed recurrence, and eight patients died within a mean follow-up time of 52 months (range, 1–96 months). The clinicopathological characteristics of these 70 patients according to BRCA protein expression status are presented in **Table 1**. The median age was 44 (range, 41~48) years for BRCA1 negative patients and 47.5 (range, 40~59.5) years for BRCA1 positive patients. Most patients were at stage IB1 (69.2% for BRCA1 negative, and 84.1% for BRCA1 positive patients). There were no significant differences in clinicopathological characteristics of patients between the two groups except for histologic cell type. Higher proportion of squamous cell carcinoma (SCC, 93.2%) was observed in the BRCA1 positive group than in the BRCA1 negative group (69.2%). Six (23.1%) patients in the BRCA1 negative group and 2 (4.5%) patients in the BRCA1 positive group died.

**Table 1:** Clinical characteristics of patients by BRCA1 protein expression.

	BRCA1 negative (n = 26)	BRCA1 positive (n = 44)	p-value
**Age, years, median (range)**	44 (41~48)	47.5 (40~59.5)	0.374
**Stage, *n* (%)**			0.245
IB1	18 (69.2)	37 (84.1)	
IIA	8 (30.8)	7 (15.9)	
**SCC Ag, ng/mL, median (range)**	1.6 (0.9~4.2)	1.6 (1.1~5.1)	0.484
**Cell type**			**0.020**
SCC	18 (69.2)	41 (93.2)	
AC	8 (30.8)	3 (6.8)	
**Size, cm, median (range)**	3.5 (2.2~5.0)	3.1 (2.1~4.5)	0.368
**Parametrial invasion, *n* (%)**			0.732
No	25 (96.2)	40 (90.9)	
Yes	1 (3.8)	4 (9.1)	
**Resection margin with tumor, *n* (%)**			0.789
No	25 (96.2)	44 (100.0)	
Yes	1(3.8)	0	
**LVSI, *n* (%)**			0.252
No	5 (33.3)	19 (55.9)	
Yes	10 (66.7)	15 (44.1)	
**Deep tumor invasion, *n* (%)**			0.288
≤1cm	14 (56.0)	17 (39.5)	
>1cm	11 (44.0)	26 (60.5)	
**Deep tumor invasion, *n* (%)**			0.193
≤cervical depth 1/2	10 (38.5)	9 (20.9)	
>cervical depth 1/2	16 (61.5)	34 (79.1)	
**LN metastasis, *n* (%)**			0.714
No	14 (53.8)	27 (61.4)	
Yes	12 (46.2)	17 (38.6)	
**Recurrence, *n* (%)**			0.288
No	19 (73.1)	38 (86.4)	
Yes	7 (26.9)	6 (13.6)	
**Death, *n* (%)**			**0.049**
No	20 (76.9)	42 (95.5)	
Yes	6 (23.1)	2 (4.5)	

SCC, Squamous cell carcinoma; AC, Adenocarcinoma; LVSI, Lympho-vascular space invasion; LN, Lymph node. Bold values mean they are statistically significant.

### BRCA IHC Expression and Prognostic Significance

**Figure 2:**
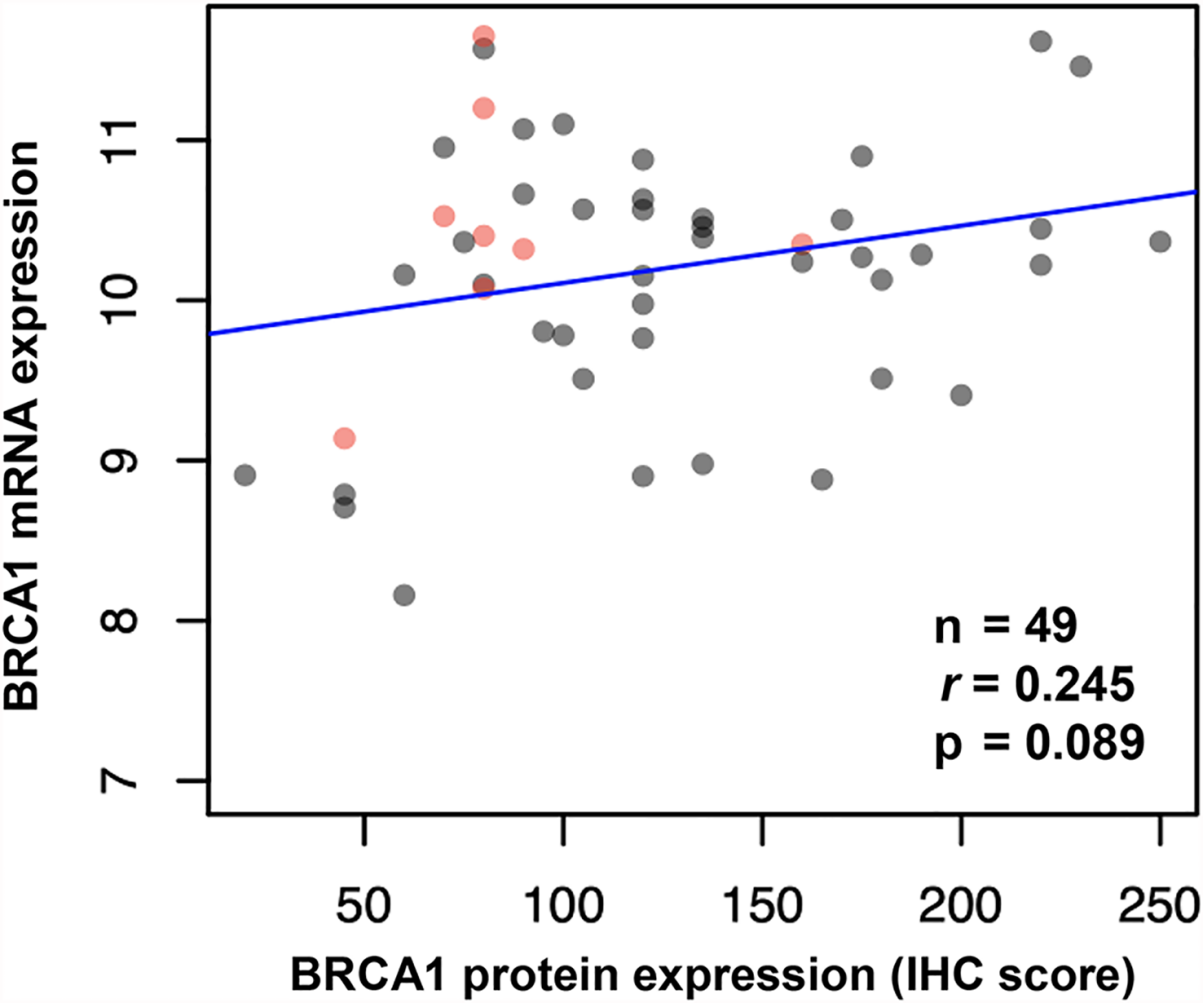
Correlation between BRCA1 mRNA and protein expression (IHC score) in cervical cancer tissues shown by Spearman’s rho coefficient. (Red dots indicate adenocarcinoma, and black dots indicate squamous cell carcinoma).

We performed immunohistochemistry staining for BRCA1 using cervical cancer tissues. BRCA1 expression was mainly seen in the cytoplasm or the nucleus. Representative examples of positive and negative staining of BRCA1 are shown in **Figure 1**. We examined correlations between BRCA1 protein and mRNA expression levels by using Spearman’s rho coefficient. There was a positive correlation (r = 0.245, p = 0.089, **Figure 2**) between the BRCA1 protein and mRNA expression levels, although the correlation was not statistically significant. In **Figure 2**, red dots indicate adenocarcinoma and black dots imply squamous cell carcinoma. Adenocarcinoma was mostly observed in area of high BRCA1 mRNA expression and low BRCA1 IHC expression. For further investigation, when patient’s overall survival information was applied (data not shown), death events also showed a tendency to be associated with high BRCA1 mRNA expression and low BRCA1 IHC expression. It can be inferred that mRNA expression and protein expression have a different association with cancer prognosis and further research is needed in this regard.

**Figure 3:**
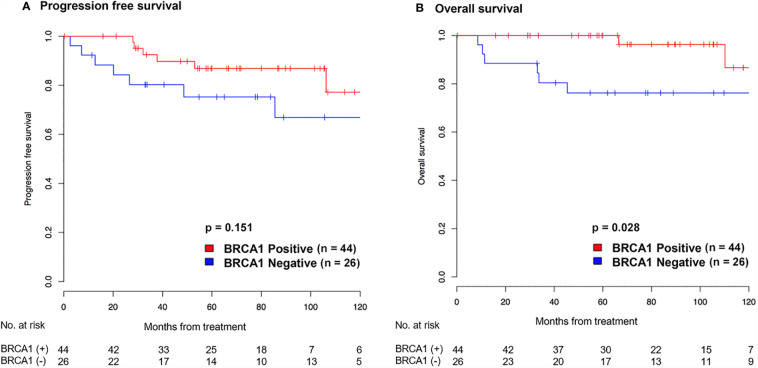
Kaplan-Meier graph showing progression-free survival **(A)** and overall survival **(B)** according to BRCA1 expression in patients with cervical cancer.

Kaplan-Meier curves of cervical cancer patients with positive or negative BRCA1 expression are shown in **Figure 3**. The BRCA1 negative group showed inferior survival outcomes, including worse PFS (**Figure 3A**) and OS (**Figure 3B**). However, significant worse survival outcome was only found for the OS (HR: 0.20, 95% CI: 0.04 – 0.99, p = 0.028), not the PFS (HR: 0.46, 95% CI: 0.15 – 1.37, p = 0.151). The prognostic significance of BRCA1 expression showed a trend in multivariate analysis (HR: 0.21, 95% CI: 0.04 – 1.11) (**Table 2**). However, it was not statistically significant (p = 0.06).

**Table 2:** Multivariate cox regression analysis for the overall survival.

	HR	95% CI	p-value
BRCA1 positive	0.20	(0.04 – 1.11)	0.066
Stage	4.12	(0.85 – 19.93)	0.078
LN metastasis	0.75	(0.15 – 3.74)	0.730
Cell type	1.86	(0.38 – 9.12)	0.442
Parametrial invasion	3.32	(0.49 – 22.45)	0.219

HR, hazard ratio; CI, confidence interval.

### Identification of Molecular Signatures Associated With BRCA1

To further examine the prognostic significance of BRCA1 expression, we analyzed the GEO database (GSE44001). Analysis of gene expression profiles by BRCA1 expression identified 320 genes whose expression levels were correlated with BRCA1 expression (correlation coefficient > 0.5 or < -0.5 and p*-*value < 0.05) (**Supplementary Table S1**). Of these, 178 genes were positively correlated while 142 genes were negatively correlated with BRCA1 expression.

Of these 320 genes, Cox regression with backward elimination was performed to select genes as independent prognostic markers. Twenty genes (PGK1, FBLN5, CCR6, CEP170, FZD1, SNORD35A, COL1A2, RECK, SNORA75, SNORA2B, SERINC5, SNORA11D, MAPK14, SNORD21, SDHAP3, TMSB4X, LAMB1, SNORD8, SCARNA5, and SNORA50A) were selected. Univariate analysis results using these selected 20 genes are shown in **Table 3**. The Kaplan-Meier survival curve is shown in **Supplementary Figure S1**.

**Table 3:** Univariate analysis for overall survival of 20 selected genes correlated with BRCA1.

	HR	95% CI	p-value
PGK1	5.42	(2.01 – 14.58)	0.001
FBLN5	0.37	(0.14 – 1)	0.049
CCR6	0.33	(0.15 – 0.72)	0.006
CEP170	0.26	(0.07 – 0.92)	0.036
FZD1	0.23	(0.03 – 1.65)	0.143
SNORD35A	0.48	(0.28 – 0.84)	0.01
COL1A2	0.08	(0.02 – 0.42)	0.002
RECK	3.49	(1.04 – 11.67)	0.042
SNORA75	0.39	(0.14 – 1.05)	0.062
SNORA2B	0.09	(0.01 – 0.61)	0.013
SERINC5	0.34	(0.09 – 1.3)	0.116
SNORA11D	2.62	(0.88 – 7.77)	0.084
MAPK14	3.57	(1.02 – 12.53)	0.047
SNORD21	0.28	(0.06 – 1.36)	0.116
SDHAP3	0.46	(0.19 – 1.14)	0.095
TMSB4X	6.79	(1.06 – 43.35)	0.043
LAMB1	3.8	(1.1 – 13.1)	0.035
SNORD8	7.31	(1.18 – 45.49)	0.033
SCARNA5	5.89	(1.12 – 30.93)	0.036
SNORA50A	5.29	(1.02 – 27.41)	0.047

HR, hazard ratio; CI, confidence interval.

### Gene Set Enrichment Analysis (GSEA)

Gene set enrichment analysis (GSEA) results showed one up-regulated pathway and one down-regulated pathway in cervical cancer (**Supplementary Table S2** and **Supplementary Figure S2**). VEGF A-regulated gene pathway was downregulated, and E2F1-regulated gene pathway was upregulated. E2F1-regulated genes modulate the transition from quiescence into DNA synthesis, or have roles in apoptosis, signal transduction, membrane biology, and transcription repression. The other dysregulated pathway without statistical significance were PRC2_EZH2_UP.V1_UP (p = 0.067), CSR_LATE_UP.V1_UP (p = 0.055), HOXA9_DN.V1_DN (p = 0.081), TBK1.DF_DN (p = 0.059), GCNP_SHH_UP_LATE.V1_UP (p = 0.214), CSR_EARLY_UP.V1_UP (p = 0.152), ERB2_UP.V1_DN (p = 0.246), TBK1.DF_UP (p = 0.599), GCNP_SHH_UP_EARLY.V1_UP (p = 0.620), CAMP_UP.V1_DN (p = 0.765), and LTE2_UP.V1_DN (p = 0.889).

## Discussion

This study was initiated with hypothesis that BRCA1 expression may be associated to prognosis in cervical cancer. In the current study, we investigated the prognostic relevance of expression of BRCA1 in cervical cancer by IHC analysis of BRCA1 in cervical cancer tissue for comparison of overall survival according to BRCA1 expression. Additional analysis was done to find genes associated with BRCA1 expression using Gene set enrichment analysis (GSEA) method. This study showed that alteration associated to BRCA1 expression was associated with survival outcomes of patients in uterine cervical cancer.

In current study, low BRCA1 expression was associated with inferior prognosis in cervical cancer. As similar results, a previous study on BRCA expression in cancer has revealed that promoter hypermethylation can inactivate genes in the Fanconi Anemia (FA)-BRCA pathway, including BRCA1 and BRCA2 (19). Narayanet et al. (20) have reported that BRCA1 promoter hypermethylation is present in 6.1% of cervical cancer patients. Promoter hypermethylation of FANCF gene could disrupt the FA-BRCA pathway, resulting in cisplatin resistance in cervical cancer (21). Interestingly, other studies have shown different results, as BRCA-deficient cells are inefficient in repairing DNA damage through homologous recombination (HR) (22, 23) and that they are more sensitive to chemotherapeutic drugs. Also, Balacescu et al. (24) have shown that overexpression of BRCA1 and BRCA2 in patients with advanced cervical cancer is associated with treatment failure. In a recent *in vitro* study, Wen et al. (25) have also suggested that BRCA1 overexpression might be a mechanism involved in the enhanced chemoradiation resistance of cervical squamous cell carcinoma. Although previous studies tried to reveal prognostic relevance of BRCA expression, these studies did not show consistent results. The reason for showing different results may be that in addition to the known relationship between gene mutation and cancer prognosis, it can be inferred that mRNA expression and protein expression have a different association with cancer prognosis caused by mechanism such as post-transcriptional modifications. Further research is needed in this regard.

Determining BRCA1 protein expression by IHC can theoretically detect BRCA1 loss-of-function tumors. Previously, studies have shown BRCA1 IHC is effective method to screen for genetic BRCA1 alterations (12, 26), and BRCA1 IHC testing has been performed for ovarian cancer with excellent correlation with survival (12, 26–29). It can be inferred that these results will be similar for uterine cervical cancer as well but further study is warranted. Currently, BRCA1 germline testing and Next Generation Sequencing (NGS) testing are available options for BRCA assessment. However, these genetic tests remain expensive and time-consuming. As the clinical significance of somatic mutations and promoter hypermethylation is further ascertained, BRCA1 IHC testing can be an effective method to identify BRCA1 expression through both genetic and epigenetic mechanisms.

In this study, GSEA result showed that VEGF A-regulated gene pathway downregulation was associated with BRCA1 expression. VEGF plays a role in tumor angiogenesis which is essential for tumor growth, invasion, and metastasis (30). However, previous studies on the relationship between angiogenesis marker and prognosis of cervical cancer have reported conflicting results. Zijlmans et al. (31) reported that VEGF mRNA expression correlated with CD31 expression, which is a marker for microvessel density (MVD) analysis, and CD31 expression was associated with lymphovascular space invasion and lymph node metastasis. With same context, a study in stage IB cervical cancer showed that patients with high tumor MVD has poor survival (5-year survival 63% *vs*. 90%) (32). These are consistent with our finding, assuming that BRCA1 positive tumors with VEGF A pathway downregulation, leading to low angiogenesis and better prognosis. On the contrary, there are reports that decreased level of tumor angiogenesis, which was represented as CD31 MVD, is associated with poor survival in high-risk, early stage cervical cancer (33, 34). They hypothesized that survival advantage in high MVD may owe to improved chemoradiation response in well-vascularized tumors. Further study is required since the relationship between tumor angiogenesis and prognosis has not yet been elucidated.

To our best knowledge, this study is first to show association of BRCA1 IHC expression and prognosis in uterine cervical cancer patients. However, this study has some limitations. First, we used a small number of samples for analysis. Second, although the relationship between IHC results and prognosis was shown, a comparative NGS study was not conducted to determine whether gene expression in cervical cancer was consistent with the BRCA1 IHC results. Previous studies have shown that IHC reflects gene alteration in ovarian cancer, but research results are still lacking in cervical cancer. Instead, we used an additional validation with microarray data for associated gene alterations but further study is needed. Also, we were not able to show a mechanism to explain the relationship between low BRCA1 expression and inferior survival in current analysis. Further studies with larger sample sizes are warranted to determine whether BRCA1 expression is an important prognostic factor that determines poor response to radiation and chemotherapy for subgroups of patients with cervical cancer.

## Conclusions

In conclusion, low expression of BRCA1 in cervical cancer has potential prognostic and therapeutic significance. Our results indicated that BRCA1 IHC may be effective method to identify BRCA1 expression through both genetic and epigenetic mechanisms to identify these patients in the clinic.

## Data Availability Statement

Publicly available datasets were analyzed in this study. This data can be found here: http://www.ncbi.nlm.nih.gov/geo/query/acc.cgi?acc=GSE44001, https://www.gsea-msigdb.org/gsea/msigdb/genesets.jsp?collection=C6.

## Ethics Statement

The studies involving human participants were reviewed and approved by the institutional review boards (IRB) of Samsung Medical Center, Seoul, Republic of Korea. The patients/participants provided their written informed consent to participate in this study.

## Author Contributions

Conceptualization, CHC and Y-YL. Methodology, CHC and S-JB. Formal Analysis, EP. Investigation, CK. Data Curation, YC and SO. Writing – Original Draft Preparation, C-SC and EP. Writing – Review & Editing, EP and C-SC. Supervision, T-JK. Funding Acquisition, J-WL and B-GK. All authors contributed to the article and approved the submitted version.

## Funding

This research was supported by a grant (2017R1D1A1B05035844) of the Basic Science Research Program through the National Research Foundation of Korea (NRF) funded by the Ministry of Education, Samsung Medical Center (SMO1210211), and a grant (NCC1810860) funded by the National Cancer Center, Republic of Korea.

## Conflict of Interest

The authors declare that the research was conducted in the absence of any commercial or financial relationships that could be construed as a potential conflict of interest.

## Publisher’s Note

All claims expressed in this article are solely those of the authors and do not necessarily represent those of their affiliated organizations, or those of the publisher, the editors and the reviewers. Any product that may be evaluated in this article, or claim that may be made by its manufacturer, is not guaranteed or endorsed by the publisher.

## Supplementary Material

Supplementary Figure 1 Kaplan-Meier graph showing overall survival of BRCA1 correlated genes.

Supplementary Figure 2 Gene Set Enrichment Analysis of cervical cancer.

## References

[1] Ferlay J Soerjomataram I Dikshit R Eser S Mathers C Rebelo M. Cancer Incidence and Mortality Worldwide: Sources, Methods and Major Patterns in GLOBOCAN 2012. Int J Cancer (2015) 136(5):E359–86. doi: 10.1002/ijc.29210 25220842

[2] Lee WC Lee SY Koo YJ Kim TJ Hur SY Hong SR. Establishment of a Korea HPV Cohort Study. J Gynecol Oncol (2013) 24(1):59–65. doi: 10.3802/jgo.2013.24.1.59 23346315 PMC3549509

[3] Delgado G Bundy B Zaino R Sevin BU Creasman WT Major F. Prospective Surgical-Pathological Study of Disease-Free Interval in Patients With Stage IB Squamous Cell Carcinoma of the Cervix: A Gynecologic Oncology Group Study. Gynecol Oncol (1990) 38(3):352–7. doi: 10.1016/0090-8258(90)90072-s 2227547

[4] Landoni F Maneo A Colombo A Placa F Milani R Perego P. Randomised Study of Radical Surgery *Versus* Radiotherapy for Stage Ib-IIa Cervical Cancer. Lancet (1997) 350(9077):535–40. doi: 10.1016/S0140-6736(97)02250-2 9284774

[5] Lippert TH Ruoff HJ Volm M. Intrinsic and Acquired Drug Resistance in Malignant Tumors. The Main Reason for Therapeutic Failure. Arzneimittelforschung (2008) 58(6):261–4. doi: 10.1055/s-0031-1296504 18677966

[6] Branzei D Foiani M. Regulation of DNA Repair Throughout the Cell Cycle. Nat Rev Mol Cell Biol (2008) 9(4):297–308. doi: 10.1038/nrm2351 18285803

[7] Birgisdottir V Stefansson OA Bodvarsdottir SK Hilmarsdottir H Jonasson JG Eyfjord JE. Epigenetic Silencing and Deletion of the BRCA1 Gene in Sporadic Breast Cancer. Breast Cancer Res (2006) 8(4):R38. doi: 10.1186/bcr1522 16846527 PMC1779478

[8] Cancer Genome Atlas Research Network. Integrated Genomic Analyses of Ovarian Carcinoma. Nature (2011) 474(7353):609–15. doi: 10.1038/nature10166 PMC3163504 21720365

[9] Tapia T Smalley SV Kohen P Munoz A Solis LM Corvalan A. Promoter Hypermethylation of BRCA1 Correlates With Absence of Expression in Hereditary Breast Cancer Tumors. Epigenetics (2008) 3(3):157–63. doi: 10.4161/epi.3.3.6387 18567944

[10] Moskwa P Buffa FM Pan Y Panchakshari R Gottipati P Muschel RJ. miR-182-Mediated Downregulation of BRCA1 Impacts DNA Repair and Sensitivity to PARP Inhibitors. Mol Cell (2011) 41(2):210–20. doi: 10.1016/j.molcel.2010.12.005 PMC3249932 21195000

[11] Garcia AI Buisson M Bertrand P Rimokh R Rouleau E Lopez BS. Down-Regulation of BRCA1 Expression by miR-146a and miR-146b-5p in Triple Negative Sporadic Breast Cancers. EMBO Mol Med (2011) 3(5):279–90. doi: 10.1002/emmm.201100136 PMC3377076 21472990

[12] Garg K Levine DA Olvera N Dao F Bisogna M Secord AA. BRCA1 Immunohistochemistry in a Molecularly Characterized Cohort of Ovarian High-Grade Serous Carcinomas. Am J Surg Pathol (2013) 37(1):138–46. doi: 10.1097/PAS.0b013e31826cabbd PMC3531916 23232854

[13] Rabiau N Dechelotte P Adjakly M Kemeny JL Guy L Boiteux JP. BRCA1, BRCA2, AR and IGF-I Expression in Prostate Cancer: Correlation Between RT-qPCR and Immunohistochemical Detection. Oncol Rep (2011) 26(3):695–702. doi: 10.3892/or.2011.1339 21667031

[14] Milner R Wombwell H Eckersley S Barnes D Warwicker J Van Dorp E. Validation of the BRCA1 Antibody MS110 and the Utility of BRCA1 as a Patient Selection Biomarker in Immunohistochemical Analysis of Breast and Ovarian Tumours. Virchows Arch (2013) 462(3):269–79. doi: 10.1007/s00428-012-1368-y 23354597

[15] Lee YY Kim TJ Kim JY Choi CH Do IG Song SY. Genetic Profiling to Predict Recurrence of Early Cervical Cancer. Gynecol Oncol (2013) 131(3):650–4. doi: 10.1016/j.ygyno.2013.10.003 24145113

[16] Choi CH Chung JY Park HS Jun M Lee YY Kim BG. Pancreatic Adenocarcinoma Up-Regulated Factor Expression Is Associated With Disease-Specific Survival in Cervical Cancer Patients. Hum Pathol (2015) 46(6):884–93. doi: 10.1016/j.humpath.2015.02.016 PMC7717069 25870121

[17] Huang da W Sherman BT Lempicki RA. Systematic and Integrative Analysis of Large Gene Lists Using DAVID Bioinformatics Resources. Nat Protoc (2009) 4(1):44–57. doi: 10.1038/nprot.2008.211 19131956

[18] Subramanian A Tamayo P Mootha VK Mukherjee S Ebert BL Gillette MA. Gene Set Enrichment Analysis: A Knowledge-Based Approach for Interpreting Genome-Wide Expression Profiles. Proc Natl Acad Sci USA (2005) 102(43):15545–50. doi: 10.1073/pnas.0506580102 PMC1239896 16199517

[19] Jones PA Baylin SB. The Fundamental Role of Epigenetic Events in Cancer. Nat Rev Genet (2002) 3(6):415–28. doi: 10.1038/nrg816 12042769

[20] Narayan G Arias-Pulido H Koul S Vargas H Zhang FF Villella J. Frequent Promoter Methylation of CDH1, DAPK, RARB, and HIC1 Genes in Carcinoma of Cervix Uteri: Its Relationship to Clinical Outcome. Mol Cancer (2003) 2:24. doi: 10.1186/1476-4598-2-24 12773202 PMC156646

[21] Narayan G Arias-Pulido H Nandula SV Basso K Sugirtharaj DD Vargas H. Promoter Hypermethylation of FANCF: Disruption of Fanconi Anemia-BRCA Pathway in Cervical Cancer. Cancer Res (2004) 64(9):2994–7. doi: 10.1158/0008-5472.can-04-0245 15126331

[22] Roy R Chun J Powell SN. BRCA1 and BRCA2: Different Roles in a Common Pathway of Genome Protection. Nat Rev Cancer (2011) 12(1):68–78. doi: 10.1038/nrc3181 22193408 PMC4972490

[23] Rigakos G Razis E. BRCAness: Finding the Achilles Heel in Ovarian Cancer. Oncologist (2012) 17(7):956–62. doi: 10.1634/theoncologist.2012-0028 PMC3399652 22673632

[24] Balacescu O Balacescu L Tudoran O Todor N Rus M Buiga R. Gene Expression Profiling Reveals Activation of the FA/BRCA Pathway in Advanced Squamous Cervical Cancer With Intrinsic Resistance and Therapy Failure. BMC Cancer (2014) 14:246. doi: 10.1186/1471-2407-14-246 24708616 PMC4021393

[25] Wen X Liu S Cui M. Effect of BRCA1 on the Concurrent Chemoradiotherapy Resistance of Cervical Squamous Cell Carcinoma Based on Transcriptome Sequencing Analysis. BioMed Res Int (2020) 2020:3598417. doi: 10.1155/2020/3598417 32685473 PMC7333031

[26] Meisel JL Hyman DM Garg K Zhou Q Dao F Bisogna M. The Performance of BRCA1 Immunohistochemistry for Detecting Germline, Somatic, and Epigenetic BRCA1 Loss in High-Grade Serous Ovarian Cancer. Ann Oncol (2014) 25(12):2372–8. doi: 10.1093/annonc/mdu461 PMC4271017 25281711

[27] Byrne TJ Reece MT Adams LA Lane MA Cohn GM. An Antibody Assay Predictive of BRCA1 Mutations in Ovarian Tumors and Normal Tissue. Oncol Rep (2000) 7(5):949–53. doi: 10.3892/or.7.5.949 10948320

[28] Skytte AB Waldstrom M Rasmussen AA Cruger D Woodward ER Kolvraa S. Identification of BRCA1-Deficient Ovarian Cancers. Acta Obstet Gynecol Scand (2011) 90(6):593–9. doi: 10.1111/j.1600-0412.2011.01121.x 21371001

[29] Vaz FH Machado PM Brandao RD Laranjeira CT Eugenio JS Fernandes AH. Familial Breast/Ovarian Cancer and BRCA1/2 Genetic Screening: The Role of Immunohistochemistry as an Additional Method in the Selection of Patients. J Histochem Cytochem (2007) 55(11):1105–13. doi: 10.1369/jhc.7A7209.2007 PMC3957528 17625228

[30] Carmeliet P Jain RK. Angiogenesis in Cancer and Other Diseases. Nature (2000) 407(6801):249–57. doi: 10.1038/35025220 11001068

[31] Zijlmans HJ Fleuren GJ Hazelbag S Sier CF Dreef EJ Kenter GG. Expression of Endoglin (CD105) in Cervical Cancer. Br J Cancer (2009) 100(10):1617–26. doi: 10.1038/sj.bjc.6605009 PMC2696762 19352388

[32] Obermair A Wanner C Bilgi S Speiser P Kaider A Reinthaller A. Tumor Angiogenesis in Stage IB Cervical Cancer: Correlation of Microvessel Density With Survival. Am J Obstet Gynecol (1998) 178(2):314–9. doi: 10.1016/s0002-9378(98)80018-5 9500492

[33] Randall LM Monk BJ Darcy KM Tian C Burger RA Liao SY. Markers of Angiogenesis in High-Risk, Early-Stage Cervical Cancer: A Gynecologic Oncology Group Study. Gynecol Oncol (2009) 112(3):583–9. doi: 10.1016/j.ygyno.2008.11.013 PMC2858218 19110305

[34] Kim YH Kim MA Park IA Park WY Kim JW Kim SC. VEGF Polymorphisms in Early Cervical Cancer Susceptibility, Angiogenesis, and Survival. Gynecol Oncol (2010) 119(2):232–6. doi: 10.1016/j.ygyno.2010.07.035 20797778

