# Supplementary figures and images for: Prognostic Relevance of BRCA1 Expression in Survival of Patients With Cervical Cancer
Source: Front Oncol. 2021 Nov 8;11:770103. doi: 10.3389/fonc.2021.770103 (PMC8606581; doi:10.3389/fonc.2021.770103)

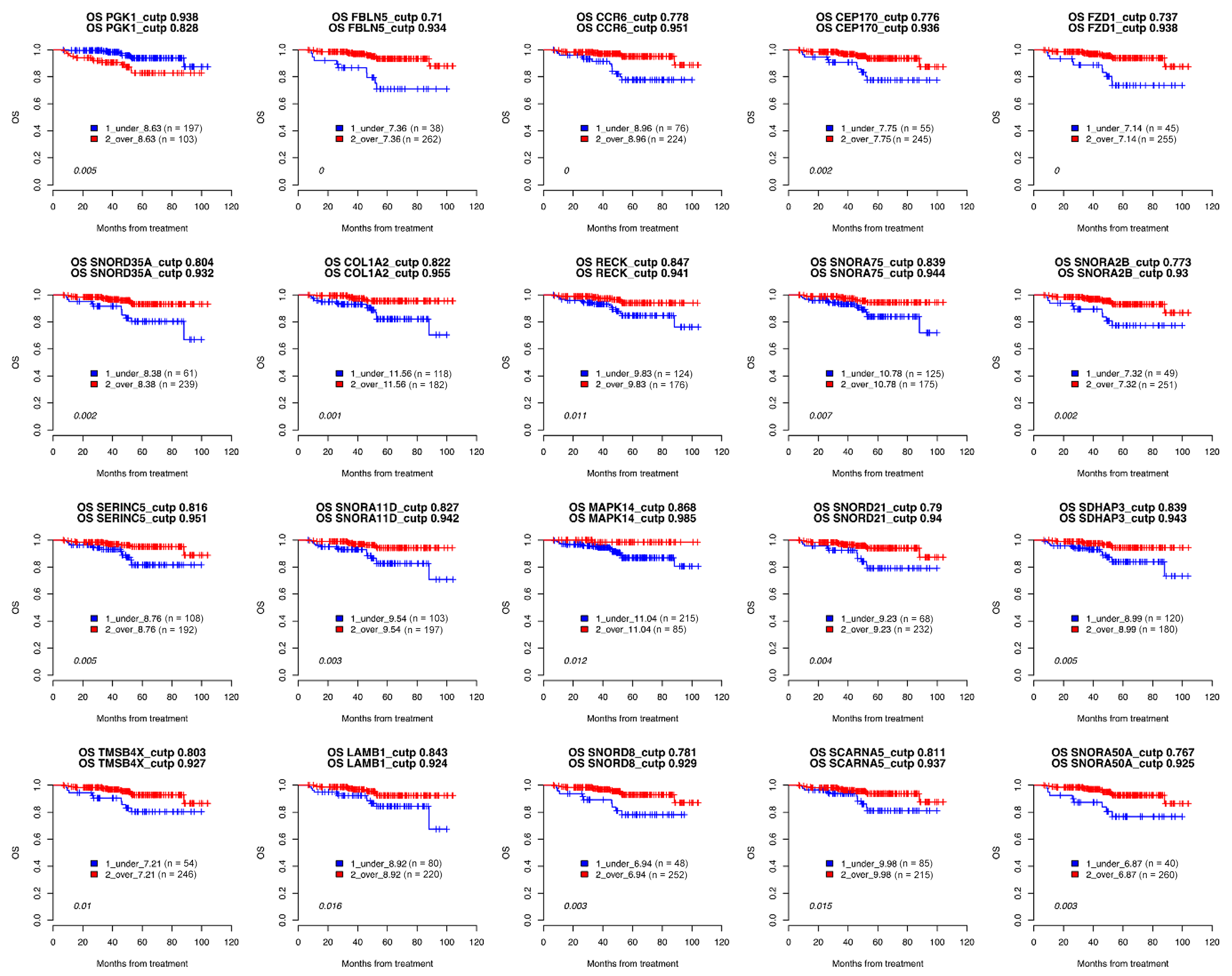

Supplement: Supplementary Figure 1 — Kaplan-Meier graph showing overall survival of BRCA1 correlated genes. [file Image_1.tif]

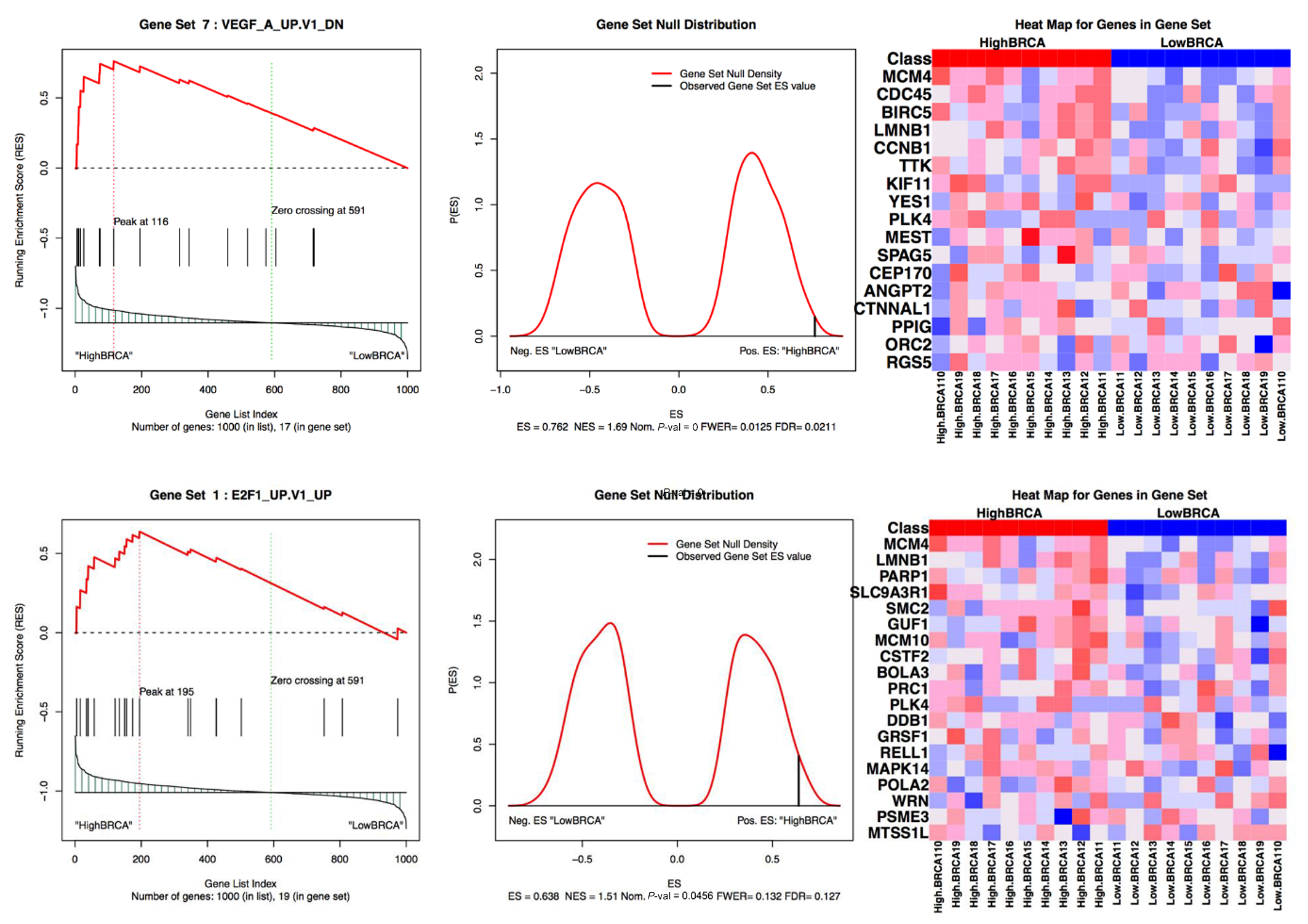

Supplement: Supplementary Figure 2 — Gene Set Enrichment Analysis of cervical cancer. [file Image_2.tif]
