# Supplementary material for: Prognostic Relevance of BRCA1 Expression in Survival of Patients With Cervical Cancer
Source: Front Oncol. 2021 Nov 8;11:770103. doi: 10.3389/fonc.2021.770103 (PMC8606581; doi:10.3389/fonc.2021.770103)
Supplement: Supplementary file 3 [file Table_1.docx]

**Table S1 |** Differentially expressed genes according to BRCA1 expression.

| ***gene*** | **R** | **P-value** | **FDR P-value** |
| --- | --- | --- | --- |
| *CIT* | 0.74371367 | 4.70E-54 | 4.60E-50 |
| *ASPM* | 0.73566603 | 2.39E-52 | 1.75E-48 |
| *KIF20B* | 0.70849453 | 5.06E-47 | 1.86E-43 |
| *CDC45* | 0.69008591 | 9.49E-44 | 2.79E-40 |
| *NR2C2AP* | 0.6882851 | 1.92E-43 | 4.35E-40 |
| *CENPM* | 0.68394234 | 1.04E-42 | 2.18E-39 |
| *SMC2* | 0.68190047 | 2.27E-42 | 4.44E-39 |
| *HELLS* | 0.65363065 | 6.11E-38 | 8.55E-35 |
| *CENPH* | 0.65297663 | 7.64E-38 | 1.02E-34 |
| *TROAP* | 0.64890587 | 3.02E-37 | 3.70E-34 |
| *FAM72D* | 0.64745039 | 4.92E-37 | 5.78E-34 |
| *NUSAP1* | 0.64584377 | 8.40E-37 | 9.14E-34 |
| *GGH* | 0.64541528 | 9.68E-37 | 9.81E-34 |
| *PARP1* | 0.64491674 | 1.14E-36 | 1.12E-33 |
| *MRPL13* | 0.64275857 | 2.32E-36 | 2.13E-33 |
| *FANCC* | 0.63996047 | 5.79E-36 | 5.15E-33 |
| *PTTG1* | 0.63428558 | 3.58E-35 | 3.09E-32 |
| *HMGB2* | 0.63375228 | 4.24E-35 | 3.56E-32 |
| *POLE3* | 0.63124354 | 9.37E-35 | 7.64E-32 |
| *DROSHA* | 0.62936997 | 1.68E-34 | 1.34E-31 |
| *R3HDM1* | 0.62793273 | 2.64E-34 | 2.04E-31 |
| *ASF1B* | 0.62295453 | 1.22E-33 | 8.33E-31 |
| *CDCA4* | 0.62197389 | 1.64E-33 | 1.10E-30 |
| *CCNB1* | 0.62157359 | 1.86E-33 | 1.21E-30 |
| *PLK1* | 0.6208673 | 2.30E-33 | 1.47E-30 |
| *MCM10* | 0.62067413 | 2.44E-33 | 1.52E-30 |
| *CCDC77* | 0.61415474 | 1.71E-32 | 1.05E-29 |
| *TMX1* | 0.61339 | 2.14E-32 | 1.29E-29 |
| *AK2* | 0.61046369 | 5.06E-32 | 2.91E-29 |
| *CSTF2* | 0.60379483 | 3.45E-31 | 1.81E-28 |
| *MRPL11* | 0.60302006 | 4.31E-31 | 2.14E-28 |
| *TUBA1B* | 0.60234029 | 5.22E-31 | 2.53E-28 |
| *COX5A* | 0.60202092 | 5.72E-31 | 2.67E-28 |
| *RAD54L* | 0.60188573 | 5.94E-31 | 2.73E-28 |
| *MND1* | 0.60066923 | 8.37E-31 | 3.73E-28 |
| *PPIH* | 0.60024866 | 9.42E-31 | 4.13E-28 |
| *LMNB2* | 0.59759133 | 1.98E-30 | 8.32E-28 |
| *CENPN* | 0.59601582 | 3.07E-30 | 1.22E-27 |
| *MRPS18B* | 0.59197119 | 9.35E-30 | 3.39E-27 |
| *ACAD10* | 0.5904521 | 1.41E-29 | 4.95E-27 |
| *KLF5* | 0.58968991 | 1.74E-29 | 5.87E-27 |
| *SLC9A3R1* | 0.58932419 | 1.92E-29 | 6.41E-27 |
| *HCCS* | 0.58891715 | 2.14E-29 | 7.08E-27 |
| *MASTL* | 0.58781065 | 2.89E-29 | 9.23E-27 |
| *YES1* | 0.58699069 | 3.60E-29 | 1.14E-26 |
| *PNPO* | 0.58505252 | 6.05E-29 | 1.81E-26 |
| *SRRT* | 0.58246037 | 1.20E-28 | 3.44E-26 |
| *POLA2* | 0.58218597 | 1.30E-28 | 3.66E-26 |
| *TXNDC9* | 0.58071206 | 1.91E-28 | 5.15E-26 |
| *KIF15* | 0.57804394 | 3.84E-28 | 9.89E-26 |
| *NEK2* | 0.57714671 | 4.85E-28 | 1.22E-25 |
| *ACBD7* | 0.57617799 | 6.23E-28 | 1.50E-25 |
| *TXNDC17* | 0.57616766 | 6.25E-28 | 1.50E-25 |
| *CCSAP* | 0.57564027 | 7.16E-28 | 1.70E-25 |
| *CENPU* | 0.57349797 | 1.24E-27 | 2.83E-25 |
| *TMEM30B* | 0.57213866 | 1.76E-27 | 3.95E-25 |
| *DNAJB11* | 0.57144635 | 2.10E-27 | 4.60E-25 |
| *FN3KRP* | 0.57126065 | 2.20E-27 | 4.79E-25 |
| *ARHGEF35* | 0.57014847 | 2.92E-27 | 6.17E-25 |
| *ST6GALNAC2* | 0.56952356 | 3.42E-27 | 7.12E-25 |
| *NUDT15* | 0.56803327 | 4.98E-27 | 1.01E-24 |
| *RTKN* | 0.56789936 | 5.15E-27 | 1.04E-24 |
| *INTS7* | 0.56703137 | 6.40E-27 | 1.26E-24 |
| *USP1* | 0.56521043 | 1.01E-26 | 1.96E-24 |
| *NDUFAB1* | 0.56443351 | 1.22E-26 | 2.32E-24 |
| *SLC25A13* | 0.5633805 | 1.59E-26 | 2.98E-24 |
| *POLQ* | 0.56165629 | 2.44E-26 | 4.39E-24 |
| *SAP30L* | 0.56054646 | 3.20E-26 | 5.59E-24 |
| *SUMO1* | 0.55960641 | 4.03E-26 | 6.92E-24 |
| *CS* | 0.55960599 | 4.03E-26 | 6.92E-24 |
| *CERS6* | 0.55879871 | 4.91E-26 | 8.28E-24 |
| *CKS1B* | 0.55819871 | 5.68E-26 | 9.42E-24 |
| *CISD1* | 0.55814147 | 5.76E-26 | 9.50E-24 |
| *CDT1* | 0.5560459 | 9.57E-26 | 1.54E-23 |
| *CASP6* | 0.55513064 | 1.19E-25 | 1.91E-23 |
| *DSCC1* | 0.55508096 | 1.21E-25 | 1.92E-23 |
| *RAD21* | 0.55496366 | 1.24E-25 | 1.96E-23 |
| *RMI1* | 0.55487452 | 1.27E-25 | 1.99E-23 |
| *E2F2* | 0.55414942 | 1.51E-25 | 2.34E-23 |
| *LIN54* | 0.55407924 | 1.54E-25 | 2.35E-23 |
| *CDC7* | 0.55363213 | 1.71E-25 | 2.59E-23 |
| *PGK1* | 0.55277495 | 2.10E-25 | 3.15E-23 |
| *PPP4R1* | 0.55182711 | 2.63E-25 | 3.93E-23 |
| *DEK* | 0.55170215 | 2.71E-25 | 4.02E-23 |
| *PPM1G* | 0.55039611 | 3.70E-25 | 5.35E-23 |
| *NA* | 0.55034931 | 3.74E-25 | 5.39E-23 |
| *ZNF672* | 0.55013 | 3.94E-25 | 5.63E-23 |
| *CDC25C* | 0.55012199 | 3.95E-25 | 5.63E-23 |
| *SRRM1* | 0.55007954 | 3.99E-25 | 5.63E-23 |
| *BTG3* | 0.5500043 | 4.06E-25 | 5.71E-23 |
| *DEPDC1B* | 0.54996258 | 4.10E-25 | 5.74E-23 |
| *CKAP2* | 0.54963268 | 4.43E-25 | 6.17E-23 |
| *DDX55* | 0.54934862 | 4.74E-25 | 6.51E-23 |
| *BOLA3* | 0.54760298 | 7.15E-25 | 9.51E-23 |
| *ACBD3* | 0.54665354 | 8.93E-25 | 1.16E-22 |
| *NIPA1* | 0.54504224 | 1.30E-24 | 1.61E-22 |
| *UBA5* | 0.54378829 | 1.74E-24 | 2.09E-22 |
| *EEF2K* | 0.5432262 | 1.98E-24 | 2.35E-22 |
| *ZDHHC5* | 0.54245105 | 2.37E-24 | 2.80E-22 |
| *ROMO1* | 0.54219088 | 2.52E-24 | 2.95E-22 |
| *CCDC59* | 0.54079005 | 3.48E-24 | 4.01E-22 |
| *CYB5B* | 0.53936922 | 4.82E-24 | 5.42E-22 |
| *SARNP* | 0.53927167 | 4.93E-24 | 5.52E-22 |
| *AIFM1* | 0.53842567 | 5.97E-24 | 6.60E-22 |
| *SLF1* | 0.5360641 | 1.02E-23 | 1.10E-21 |
| *TTK* | 0.53567149 | 1.12E-23 | 1.20E-21 |
| *ERBB2* | 0.53551238 | 1.16E-23 | 1.24E-21 |
| *WSB2* | 0.53548774 | 1.16E-23 | 1.24E-21 |
| *KIF2C* | 0.53512419 | 1.26E-23 | 1.33E-21 |
| *NETO2* | 0.53492791 | 1.32E-23 | 1.39E-21 |
| *MGA* | 0.53456964 | 1.43E-23 | 1.49E-21 |
| *UBAC2* | 0.5345258 | 1.44E-23 | 1.50E-21 |
| *TNFRSF10B* | 0.53429099 | 1.52E-23 | 1.57E-21 |
| *GCNT2* | 0.5310285 | 3.16E-23 | 3.12E-21 |
| *SNAPIN* | 0.53101588 | 3.16E-23 | 3.12E-21 |
| *CDKN2A* | 0.53089514 | 3.25E-23 | 3.18E-21 |
| *NAA60* | 0.53084994 | 3.28E-23 | 3.20E-21 |
| *AP1B1* | 0.53022249 | 3.77E-23 | 3.66E-21 |
| *RNASEH2A* | 0.53009558 | 3.88E-23 | 3.73E-21 |
| *HEATR6* | 0.52847878 | 5.55E-23 | 5.22E-21 |
| *ZCCHC10* | 0.52827909 | 5.80E-23 | 5.39E-21 |
| *C6orf47* | 0.52808421 | 6.05E-23 | 5.56E-21 |
| *TRAF3IP2* | 0.52808257 | 6.05E-23 | 5.56E-21 |
| *SDHC* | 0.52765676 | 6.65E-23 | 5.99E-21 |
| *AUNIP* | 0.52740582 | 7.02E-23 | 6.29E-21 |
| *DENND1B* | 0.52714572 | 7.44E-23 | 6.64E-21 |
| *CC2D1B* | 0.5260452 | 9.46E-23 | 8.12E-21 |
| *FANCI* | 0.52603681 | 9.48E-23 | 8.12E-21 |
| *STK24* | 0.52542964 | 1.08E-22 | 9.25E-21 |
| *ILDR1* | 0.52525475 | 1.12E-22 | 9.55E-21 |
| *MTX1* | 0.52300138 | 1.83E-22 | 1.50E-20 |
| *LGALS8* | 0.52285864 | 1.89E-22 | 1.54E-20 |
| *ZNRF2* | 0.5225692 | 2.01E-22 | 1.62E-20 |
| *PSME3* | 0.52181639 | 2.37E-22 | 1.89E-20 |
| *RPA2* | 0.52168246 | 2.44E-22 | 1.94E-20 |
| *SLC1A5* | 0.52131358 | 2.64E-22 | 2.09E-20 |
| *C12orf65* | 0.51990844 | 3.57E-22 | 2.76E-20 |
| *AURKA* | 0.517652 | 5.78E-22 | 4.28E-20 |
| *PERP* | 0.51759242 | 5.85E-22 | 4.32E-20 |
| *ZDHHC23* | 0.51694205 | 6.72E-22 | 4.91E-20 |
| *RNF114* | 0.51686625 | 6.83E-22 | 4.98E-20 |
| *IMMT* | 0.51633294 | 7.65E-22 | 5.49E-20 |
| *SCYL2* | 0.51589753 | 8.38E-22 | 5.96E-20 |
| *CKAP2L* | 0.51444011 | 1.14E-21 | 7.94E-20 |
| *UBA6* | 0.5140847 | 1.23E-21 | 8.49E-20 |
| *YWHAG* | 0.51405688 | 1.24E-21 | 8.52E-20 |
| *SCRIB* | 0.51357207 | 1.37E-21 | 9.30E-20 |
| *BAG5* | 0.51322413 | 1.47E-21 | 9.96E-20 |
| *TRIM7* | 0.51268448 | 1.65E-21 | 1.10E-19 |
| *DNASE2* | 0.51231669 | 1.78E-21 | 1.18E-19 |
| *CYREN* | 0.51202956 | 1.89E-21 | 1.24E-19 |
| *MCUB* | 0.51190667 | 1.94E-21 | 1.27E-19 |
| *TIMM17B* | 0.51136703 | 2.17E-21 | 1.40E-19 |
| *ZNF146* | 0.51117805 | 2.26E-21 | 1.45E-19 |
| *DIS3L* | 0.51081328 | 2.43E-21 | 1.55E-19 |
| *MAPKAPK5* | 0.50957557 | 3.15E-21 | 1.95E-19 |
| *TRIP13* | 0.50955325 | 3.16E-21 | 1.96E-19 |
| *GLMN* | 0.50913709 | 3.45E-21 | 2.12E-19 |
| *MYO10* | 0.50878421 | 3.71E-21 | 2.27E-19 |
| *SMG5* | 0.50844619 | 3.97E-21 | 2.43E-19 |
| *OSTC* | 0.50805388 | 4.31E-21 | 2.63E-19 |
| *NUP107* | 0.50783528 | 4.51E-21 | 2.73E-19 |
| *CEP83* | 0.50751143 | 4.82E-21 | 2.90E-19 |
| *DDX28* | 0.50714369 | 5.20E-21 | 3.11E-19 |
| *PSMC5* | 0.50685666 | 5.51E-21 | 3.28E-19 |
| *SDHAF3* | 0.50632699 | 6.14E-21 | 3.64E-19 |
| *MTFP1* | 0.50602064 | 6.54E-21 | 3.86E-19 |
| *PTRHD1* | 0.50549095 | 7.29E-21 | 4.27E-19 |
| *CPSF2* | 0.50511059 | 7.88E-21 | 4.57E-19 |
| *COP1* | 0.50505614 | 7.96E-21 | 4.62E-19 |
| *PGAP2* | 0.5029129 | 1.23E-20 | 6.98E-19 |
| *NR2F6* | 0.50284423 | 1.25E-20 | 7.04E-19 |
| *PRDX4* | 0.50260377 | 1.31E-20 | 7.32E-19 |
| *MID1IP1* | 0.50232991 | 1.39E-20 | 7.68E-19 |
| *TDP2* | 0.50217451 | 1.43E-20 | 7.89E-19 |
| *GON4L* | 0.50185141 | 1.53E-20 | 8.38E-19 |
| *CYB5R2* | 0.50105239 | 1.79E-20 | 9.72E-19 |
| *NUP62CL* | 0.50035223 | 2.06E-20 | 1.10E-18 |
| *CD84* | -0.5004835 | 2.01E-20 | 1.08E-18 |
| *TMSB4X* | -0.5006966 | 1.93E-20 | 1.03E-18 |
| *EAPP* | -0.5009446 | 1.83E-20 | 9.89E-19 |
| *TPM1* | -0.5013383 | 1.69E-20 | 9.19E-19 |
| *SNORD1A* | -0.5014199 | 1.66E-20 | 9.06E-19 |
| *IGF1* | -0.5017527 | 1.56E-20 | 8.50E-19 |
| *SNORA74B* | -0.5023595 | 1.38E-20 | 7.65E-19 |
| *SERINC5* | -0.5024783 | 1.34E-20 | 7.50E-19 |
| *SNORA71E* | -0.5026918 | 1.29E-20 | 7.22E-19 |
| *SNORA73A* | -0.5027322 | 1.28E-20 | 7.17E-19 |
| *HINT3* | -0.503081 | 1.19E-20 | 6.78E-19 |
| *SNORD35B* | -0.504388 | 9.13E-21 | 5.26E-19 |
| *TIMP2* | -0.5051805 | 7.77E-21 | 4.52E-19 |
| *NA* | -0.5057746 | 6.88E-21 | 4.04E-19 |
| *COL1A2* | -0.5058724 | 6.74E-21 | 3.97E-19 |
| *FBLN1* | -0.5064923 | 5.94E-21 | 3.52E-19 |
| *SNORA75* | -0.5065814 | 5.83E-21 | 3.47E-19 |
| *CXorf36* | -0.5070226 | 5.33E-21 | 3.19E-19 |
| *SNHG15* | -0.5079456 | 4.41E-21 | 2.68E-19 |
| *DSEL* | -0.5089114 | 3.61E-21 | 2.22E-19 |
| *CEP170* | -0.5093899 | 3.27E-21 | 2.02E-19 |
| *RECK* | -0.5096848 | 3.08E-21 | 1.91E-19 |
| *RPS3* | -0.5102251 | 2.75E-21 | 1.72E-19 |
| *SNORA36B* | -0.5102972 | 2.71E-21 | 1.70E-19 |
| *RPL10A* | -0.510494 | 2.60E-21 | 1.64E-19 |
| *S1PR1* | -0.5107229 | 2.48E-21 | 1.57E-19 |
| *SCARNA2* | -0.5107735 | 2.45E-21 | 1.56E-19 |
| *SNORD41* | -0.5111262 | 2.28E-21 | 1.46E-19 |
| *SNORD15A* | -0.5111432 | 2.27E-21 | 1.46E-19 |
| *SNORD84* | -0.5111869 | 2.25E-21 | 1.45E-19 |
| *SNORD8* | -0.5114517 | 2.13E-21 | 1.38E-19 |
| *SNORA18* | -0.5121467 | 1.84E-21 | 1.21E-19 |
| *GADD45A* | -0.5130935 | 1.51E-21 | 1.02E-19 |
| *CCR6* | -0.5131064 | 1.51E-21 | 1.02E-19 |
| *RRP7BP* | -0.5135861 | 1.36E-21 | 9.30E-20 |
| *PRNP* | -0.5138332 | 1.30E-21 | 8.89E-20 |
| *SPATS2* | -0.514009 | 1.25E-21 | 8.59E-20 |
| *FOXN3* | -0.5143882 | 1.15E-21 | 8.00E-20 |
| *SNORA50A* | -0.514544 | 1.12E-21 | 7.80E-20 |
| *LMOD1* | -0.5146796 | 1.08E-21 | 7.60E-20 |
| *SDHAP3* | -0.5154507 | 9.21E-22 | 6.51E-20 |
| *CREB1* | -0.515703 | 8.74E-22 | 6.18E-20 |
| *SNORA14B* | -0.5161715 | 7.91E-22 | 5.66E-20 |
| *ISLR* | -0.516193 | 7.88E-22 | 5.64E-20 |
| *ZNF134* | -0.5163897 | 7.56E-22 | 5.44E-20 |
| *NA* | -0.5165166 | 7.35E-22 | 5.31E-20 |
| *CAPN7* | -0.5165892 | 7.24E-22 | 5.24E-20 |
| *SNORD93* | -0.5169872 | 6.66E-22 | 4.88E-20 |
| *SNORA81* | -0.5170643 | 6.55E-22 | 4.81E-20 |
| *ZNF75D* | -0.5179211 | 5.46E-22 | 4.07E-20 |
| *INO80D* | -0.5188997 | 4.43E-22 | 3.32E-20 |
| *SNORD100* | -0.5189741 | 4.36E-22 | 3.28E-20 |
| *CYP2U1* | -0.5192928 | 4.07E-22 | 3.08E-20 |
| *RBM3* | -0.5193772 | 4.00E-22 | 3.04E-20 |
| *MIR601* | -0.5196645 | 3.76E-22 | 2.87E-20 |
| *LOC154761* | -0.5198197 | 3.64E-22 | 2.80E-20 |
| *SNORA2B* | -0.5200048 | 3.50E-22 | 2.71E-20 |
| *C1R* | -0.5204383 | 3.19E-22 | 2.49E-20 |
| *SNORA47* | -0.5204625 | 3.17E-22 | 2.48E-20 |
| *SNORD96A* | -0.5205675 | 3.10E-22 | 2.44E-20 |
| *SNORD83B* | -0.5210117 | 2.82E-22 | 2.22E-20 |
| *RPL22* | -0.5212933 | 2.65E-22 | 2.09E-20 |
| *SNORA21* | -0.5218834 | 2.34E-22 | 1.87E-20 |
| *SCARNA10* | -0.52264 | 1.98E-22 | 1.60E-20 |
| *GNA12* | -0.5226551 | 1.98E-22 | 1.60E-20 |
| *SCAND2P* | -0.522859 | 1.89E-22 | 1.54E-20 |
| *SNORD21* | -0.5233615 | 1.70E-22 | 1.39E-20 |
| *RBM48* | -0.5237316 | 1.57E-22 | 1.30E-20 |
| *SNORD73A* | -0.5240108 | 1.47E-22 | 1.23E-20 |
| *ID2* | -0.5241164 | 1.44E-22 | 1.20E-20 |
| *FZD1* | -0.5241448 | 1.43E-22 | 1.20E-20 |
| *SNORD10* | -0.5253784 | 1.09E-22 | 9.32E-21 |
| *SNORA20* | -0.526246 | 9.06E-23 | 7.85E-21 |
| *RAB43* | -0.526288 | 8.97E-23 | 7.80E-21 |
| *ZNF106* | -0.5263412 | 8.87E-23 | 7.73E-21 |
| *POSTN* | -0.5264146 | 8.73E-23 | 7.63E-21 |
| *RPL26* | -0.5267843 | 8.05E-23 | 7.08E-21 |
| *GABPB1-AS1* | -0.5268913 | 7.86E-23 | 6.94E-21 |
| *SNORA49* | -0.527053 | 7.59E-23 | 6.74E-21 |
| *SNORA48* | -0.5276914 | 6.60E-23 | 5.96E-21 |
| *SNORD97* | -0.5280073 | 6.15E-23 | 5.62E-21 |
| *RNF216* | -0.5281305 | 5.99E-23 | 5.53E-21 |
| *RPS8* | -0.5284111 | 5.63E-23 | 5.26E-21 |
| *SNORA9* | -0.528456 | 5.58E-23 | 5.23E-21 |
| *SNORA68* | -0.5285957 | 5.41E-23 | 5.11E-21 |
| *SOCS5* | -0.5289684 | 4.98E-23 | 4.72E-21 |
| *NA* | -0.5297358 | 4.20E-23 | 4.01E-21 |
| *SNORD6* | -0.5300765 | 3.90E-23 | 3.73E-21 |
| *FAM126A* | -0.5309083 | 3.24E-23 | 3.18E-21 |
| *FAM53C* | -0.5312974 | 2.97E-23 | 2.96E-21 |
| *SNORA74A* | -0.5316189 | 2.77E-23 | 2.76E-21 |
| *SNORD95* | -0.5317203 | 2.71E-23 | 2.71E-21 |
| *SNORD90* | -0.5322031 | 2.43E-23 | 2.44E-21 |
| *RPS9* | -0.5322048 | 2.43E-23 | 2.44E-21 |
| *MAPK14* | -0.5329717 | 2.05E-23 | 2.09E-21 |
| *SCARNA17* | -0.5339142 | 1.66E-23 | 1.70E-21 |
| *NAIF1* | -0.5346305 | 1.41E-23 | 1.47E-21 |
| *SNORD57* | -0.5354296 | 1.18E-23 | 1.25E-21 |
| *LAMB1* | -0.5361351 | 1.00E-23 | 1.09E-21 |
| *RPS25* | -0.537704 | 7.04E-24 | 7.72E-22 |
| *SCARNA7* | -0.5388662 | 5.40E-24 | 6.01E-22 |
| *SNORD22* | -0.5392171 | 4.99E-24 | 5.57E-22 |
| *TCEAL1* | -0.5435685 | 1.83E-24 | 2.19E-22 |
| *UBB* | -0.54382 | 1.73E-24 | 2.08E-22 |
| *TFAMP1* | -0.5438527 | 1.72E-24 | 2.07E-22 |
| *SNORA3A* | -0.5439413 | 1.68E-24 | 2.05E-22 |
| *AHNAK* | -0.5444729 | 1.49E-24 | 1.83E-22 |
| *SNORD38A* | -0.5450907 | 1.29E-24 | 1.61E-22 |
| *RHOJ* | -0.5451959 | 1.26E-24 | 1.58E-22 |
| *SNORD35A* | -0.5461034 | 1.02E-24 | 1.30E-22 |
| *NA* | -0.547362 | 7.57E-25 | 1.00E-22 |
| *SNORD34* | -0.548159 | 6.28E-25 | 8.42E-23 |
| *RPL30* | -0.5492932 | 4.80E-25 | 6.56E-23 |
| *SNORD60* | -0.5495763 | 4.49E-25 | 6.22E-23 |
| *CD276* | -0.5501028 | 3.97E-25 | 5.63E-23 |
| *SNORA60* | -0.5506256 | 3.50E-25 | 5.12E-23 |
| *SNORA11D* | -0.5540857 | 1.53E-25 | 2.35E-23 |
| *SNORA77* | -0.5572299 | 7.18E-26 | 1.17E-23 |
| *SOCS3* | -0.5579635 | 6.01E-26 | 9.87E-24 |
| *SCARNA5* | -0.5610462 | 2.83E-26 | 5.01E-24 |
| *SNHG7* | -0.5618602 | 2.32E-26 | 4.20E-24 |
| *FBLN5* | -0.5625441 | 1.96E-26 | 3.61E-24 |
| *MYL9* | -0.5634326 | 1.57E-26 | 2.96E-24 |
| *SCARNA6* | -0.5646698 | 1.16E-26 | 2.23E-24 |
| *ANTXR1* | -0.5660113 | 8.27E-27 | 1.62E-24 |
| *SNORA64* | -0.5705108 | 2.66E-27 | 5.67E-25 |
| *PDZRN3* | -0.5716268 | 2.00E-27 | 4.43E-25 |
| *SNORD68* | -0.5719393 | 1.85E-27 | 4.12E-25 |
| *SYNC* | -0.5726748 | 1.53E-27 | 3.47E-25 |
| *MAGEH1* | -0.5779429 | 3.94E-28 | 1.01E-25 |
| *SNORA11D* | -0.5796781 | 2.51E-28 | 6.57E-26 |
| *CALD1* | -0.5810572 | 1.74E-28 | 4.75E-26 |
| *SNORD91B* | -0.581342 | 1.62E-28 | 4.49E-26 |
| *RBPMS* | -0.5840702 | 7.86E-29 | 2.29E-26 |
| *SNORA11* | -0.5855811 | 5.26E-29 | 1.59E-26 |
| *LBH* | -0.5880583 | 2.70E-29 | 8.73E-27 |
| *PPP1R12B* | -0.5903331 | 1.46E-29 | 5.05E-27 |
| *PRRX1* | -0.592977 | 7.10E-30 | 2.65E-27 |
| *CALD1* | -0.6033432 | 3.93E-31 | 1.99E-28 |
| *MIR645* | -0.6042048 | 3.07E-31 | 1.64E-28 |
| *KLHL28* | -0.6123352 | 2.92E-32 | 1.72E-29 |
| *ITPK1-AS1* | -0.6456078 | 9.08E-37 | 9.53E-34 |

*R, correlation coefficient; FDR, false discovery rate.*

**Table S2** | Dysregulated pathways identified in cervical cancer using Gene Set Enrichment Analysis (GSEA).

| **Gene Set** | **Description** | **SOURCE** | **ES** | **FDR ^q^-value** | **NOM P-value** |
| --- | --- | --- | --- | --- | --- |
| VEGF_A_UP.V1_DN | HUVECs (human umbilical cord vein endothelial cells) are treated with the angiogenic factor VEGF-A in low or high serum media. | http://www.broadinstitute.org/gsea/msigdb/cards/VEGF_A_UP.V1_DN | 0.76 | 0.02 | **<0.001** |
| E2F1_UP.V1_UP | Identification of E2F1-regulated genes that modulate the transition from quiescence into DNA synthesis, or have roles in apoptosis, signal transduction, membrane biology, and transcription repression. | http://www.broadinstitute.org/gsea/msigdb/cards/E2F1_UP.V1_UP | 0.64 | 0.13 | **0.045** |
| PRC2_EZH2_UP.V1_UP | The identification of gene expression changes in human embryonic fibroblast cells depleted of EZH2. | http://www.broadinstitute.org/gsea/msigdb/cards/PRC2_EZH2_UP.V1_UP | 0.66 | 0.15 | 0.067 |
| CSR_LATE_UP.V1_UP | Foreskin fibroblasts CRL 2091 (ATCC) were serum starved for 48 hours, and harvested at the indicated time points after switching to media with 10% FBS essentially as described (Iyer et al., 1999). RNA from all of the sampled time points were pooled as reference RNA to compare with RNA from individual time points as described (Iyer et al., 1999) | http://www.broadinstitute.org/gsea/msigdb/cards/CSR_LATE_UP.V1_UP | 0.67 | 0.12 | 0.055 |
| HOXA9_DN.V1_DN | RNA was purified from t(9;11) MOLM-14 AML cells 44h after transduction in triplicates with 2 of the two most effective HOXA9shRNA constructs (3 x 1F3-HOXA9shRNA; 3 x 2A5-HOXA9shRNA) or GFP-controlshRNA (6x). | http://www.broadinstitute.org/gsea/msigdb/cards/HOXA9_DN.V1_DN | 0.62 | 0.15 | 0.081 |
| TBK1.DF_DN | Genes down-regulated in epithelial lung cancer cell lines upon over-expression of an oncogenic form of KRAS [GeneID=3845] gene and knockdown of TBK1 [GeneID=29110] gene by RNAi. | http://www.broadinstitute.org/gsea/msigdb/cards/TBK1.DF_DN | 0.51 | 0.16 | 0.059 |
| GCNP_SHH_UP_LATE.V1_UP | Genes up-regulated in granule cell neuron precursors (GCNPs) after stimulation with Shh for 24h. | http://www.broadinstitute.org/gsea/msigdb/cards/GCNP_SHH_UP_LATE.V1_UP | 0.63 | 0.24 | 0.214 |
| CSR_EARLY_UP.V1_UP | Foreskin fibroblasts CRL 2091 (ATCC) were serum starved for 48 hours, and harvested at the indicated time points after switching to media with 10% FBS essentially as described (Iyer et al., 1999). RNA from all of the sampled time points were pooled as reference RNA to compare with RNA from individual time points as described (Iyer et al., 1999) | http://www.broadinstitute.org/gsea/msigdb/cards/CSR_EARLY_UP.V1_UP | 0.52 | 0.22 | 0.152 |
| ERB2_UP.V1_DN | Profiling of MCF-7 cell lines stably overexpressing constitutively active c-erbB-2 as well as control vector transfected cells (coMCF-7) and control vector transfected cells long-term adapted for estrogen-independent growth (coMCF-7/lt-E2). | http://www.broadinstitute.org/gsea/msigdb/cards/ERB2_UP.V1_DN | 0.42 | 0.31 | 0.246 |
| TBK1.DF_UP | Genes up-regulated in epithelial lung cancer cell lines upon over-expression of an oncogenic form of KRAS [GeneID=3845] gene and knockdown of TBK1 [GeneID=29110] gene by RNAi. | <http://www.broadinstitute.org/gsea/msigdb/cards/TBK1.DF_UP> | 0.42 | 0.62 | 0.599 |
| GCNP_SHH_UP_EARLY.V1_UP | Genes up-regulated in granule cell neuron precursors (GCNPs) after stimulation with Shh for 3h. | http://www.broadinstitute.org/gsea/msigdb/cards/GCNP_SHH_UP_EARLY.V1_UP | 0.35 | 0.63 | 0.620 |
| CAMP_UP.V1_DN | Genes down-regulated in primary thyrocyte cultures in response to cAMP signaling pathway activation by thyrotropin (TSH). | http://www.broadinstitute.org/gsea/msigdb/cards/CAMP_UP.V1_DN | 0.30 | 0.71 | 0.765 |
| LTE2_UP.V1_DN | Profiling of control vector transfected cells (coMCF-7) and control vector transfected cells long-term adapted for estrogen-independent growth (coMCF-7/lt-E2). | http://www.broadinstitute.org/gsea/msigdb/cards/LTE2_UP.V1_DN | 0.26 | 0.79 | 0.889 |

*ES, enrichment score; FDR, false discovery rate; NOM P-value, nominal P-value.*

ES: Enrichment score for the gene set; that is, the degree to which this gene set is overrepresented at the top or bottom of the ranked list of genes in the expression dataset.

NOM P-value: Nominal P-value; that is, the statistical significance of the enrichment score. The nominal P*-*value is not adjusted for gene set size or multiple hypothesis testing; therefore, it is of limited use in comparing gene sets.

FDR *q*-value: False discovery rate; that is, the estimated probability that the normalized enrichment score represents a false positive finding.
